# Determining Johnson-Cook Constitutive Equation for Low-Carbon Steel via Taylor Anvil Test

**DOI:** 10.3390/ma14174821

**Published:** 2021-08-25

**Authors:** Lenka Kunčická, Miroslav Jopek, Radim Kocich, Karel Dvořák

**Affiliations:** 1 Institute of Physics of Materials, Czech Academy of Sciences, Žižkova 22, 616 00 Brno, Czech Republic; 2 Department of Metal Forming and Plastic, Faculty of Mechanical Engineering, Brno University of Technology, Technická 2, 616 69 Brno, Czech Republic; m.jopek@fme.vutbr.cz; 3 Faculty of Materials Science and Technology, VŠB–Technical University of Ostrava, 708 00 Ostrava, Czech Republic; radim.kocich@vsb.cz; 4 Faculty of Civil Engineering, Institute of Technology of Building Materials and Components, Brno University of Technology, Veveří 331/95, 602 00 Brno, Czech Republic; dvorak.k@fce.vutbr.cz

**Keywords:** low-carbon steel, Taylor anvil test, Johnson–Cook, constitutive equation, high strain rate

## Abstract

Tristal steel is low-carbon construction-type steel widely used in the automotive industry, e.g., for braking components. Given the contemporary demands on the high-volume production of such components, these are typically fabricated using automatic sequential machines, which can produce components at strain rates up to 10^3^ s^−1^. For this reason, characterising the behaviour of the used material at high strain rates is of the utmost importance for successful industrial production. This study focuses on the characterisation of the behaviour of low-carbon steel via developing its material model using the Johnson-Cook constitutive equation. At first, the Taylor anvil test is performed. Subsequently, the acquired data together with the results of observations of structures and properties of the tested specimens are used to fill the necessary parameters into the equation. Finally, the developed equation is used to numerically simulate the Taylor anvil test and the predicted data is correlated with the experimentally acquired one. The results showed a satisfactory correlation of the experimental and predicted data; the deformed specimen region featured increased occurrence of dislocations, as well as higher hardness (its original value of 88 HV increased to more than 200 HV after testing), which corresponded to the predicted distributions of effective imposed strain and compressive stress.

## 1. Introduction

The primary focus in the field of bulk-forming in the automotive industry is on enhancing the performance of processing technologies and their optimisation towards higher speeds and increased productivity. For serial production, modern automatic sequential machines, which can produce components with production rates of 300 to 600 pieces per minute, are very popular [1]. However, the strain rates on the materials reach up to 10^3^ s^−1^. In recent years, high-speed forming has been a widely used room-temperature plastic deformation technology for manufacturing thin-walled and complex-shaped components [2,3,4], as well as for the production of modern types of materials, such as laminates, composites, and hybrid materials [5,6,7]. Processes of plastic forming characterised by dynamic plastic deformations at (ultra) high strain rates from 10^2^ s^−1^ to 10^6^ s^−1^, such as high-speed forming (e.g., rotary swaging), impact hydroforming, pneumo-mechanical forming, electromagnetic forming, etc., have also become very popular [8,9,10,11,12]. Moreover, high-speed deformation is the basis of numerous tests investigating the performance of manufactured components (explosive and ballistic testing, armour crashworthiness tests, etc.).

The processes featuring (ultra) high strain rates are dynamic; hardening behaviours of metallic materials under the conditions of dynamic loading differ significantly from their behaviours under the conditions of static loading. Given this fact, the dynamic behaviour of the processed materials needs to be investigated beforehand to avoid unnecessary trouble during subsequent production. The effects of dynamic loading can best be characterised by the strain rates, i.e., by quasi-static, low, intermediate, high, and ultra-high strain rates. However, the sole values of the strain rate and the loading force do not enable complex determination of the dynamic effects on the material, as they do not involve the effects of the most important parameters, characteristic for this type of loading, i.e., the elastic compressive pulse and inertial forces, which are specific for each material type.

Numerical simulations and finite element modelling in particular, are favourable tools not only to design effective structures able to support high dynamic loads but also to construct dynamic hardening models and predict material hardening behaviours at (ultra) high strain rates [13]. This fact makes them indispensable to perform the complex characterisation of dynamic plastic deformation processes. Numerous dynamic hardening material models have been developed to characterise the stress-strain relations for various materials at varying temperatures and strain rates. Among the most popular dynamic hardening material models are the Johnson–Cook model [14], the modified Johnson–Cook model [15], Khan–Huang model [16], modified Khan–Huang model [17], Zerilli–Armstrong model [18], or Preston–Tonks–Wallace (PTW) model [19].

The necessary condition to develop a mathematical description of a material model is to have performed real-time laboratory simulations under authentic time, temperature, and material conditions, the outputs of which are the basic variables (i.e., load, flow stress, strain, temperature). However, testing at ultra-high strain rates is quite a challenge limited by the available equipment. Among the methods to acquire hardening behaviours at ultra-high strain rates is extrapolation of stress–strain curves from conventional experimental results using optimised dynamic hardening models. Nevertheless, numerical simulations using the extrapolated data exhibit large deviations with the experimental results acquired, e.g., using Taylor impact tests (or also Taylor anvil tests (TAT)).

TAT has been successfully used to characterise the hardening behaviours of numerous alloys at ultra-high strain rates before. Piao et al. [20], used TAT to develop the modified Lim–Huh hardening model, which also involved the thermal effects of the deformation at a high strain rate, for AISI 4340 steel. Asala et al. [21], compared the behaviours of two different Inconel alloys during compressive deformation at high strain rates and temperatures. Others used TAT to characterise the effects of high strain rates and high temperatures on the behaviours of additively manufactured Inconels [22,23]. A few researchers also studied the static, quasi-static, and compressive mechanical behaviours of maraging steel parts manufactured by conventional forming processes [24,25], as well as via additive manufacturing [25,26]. The majority of the conducted studies only focused on the macroscopic features or investigated the behaviour of maraging steels at a limited range of strain rates. Nevertheless, Song et al. [25] used the Kolsky compression bar test to analyse the compressive behaviour of C250 maraging steel at varying strain rates and reported an increase in the dynamic strength with increasing strain rate, Schnitzer et al. [25] documented that the strain rate sensitivity decreased with increasing strain for aged maraging steels (effect of dislocation pinning on precipitates). Last but not least, Dehgahi et al. [26], investigated the behaviours of as-built and heat-treated additively manufactured maraging steel samples and found adiabatic shear bands in their structures (an effect of thermomechanical instability during dynamic processing). For the as-built samples, the adiabatic shear bands occurred at strain rates between 1500 and 3500 s^−1^, while for the heat-treated samples it was at the strain rate of 890 s^−1^. They also documented the fracture behaviours of the samples and related it to the occurrence of precipitates causing brittleness; for the as-built samples, the fracture occurred at the strain rate of 3500 s^−1^, whereas for the heat-treated ones, it was at the strain rate of 1930 s^−1^.

Despite the fact that numerous works documenting the high strain rate behaviours of various steels and alloys have been published, the majority of such studies only focused on concrete processing conditions and the type of deformation process. However, the ever-increasing selection of deformation techniques and processing technologies put increasing demands on the characterisation of the behaviours of materials at (ultra) high strain rates. For this reason, the focus of this study was to perform a complex characterisation of the ultra-high strain rate behaviour of TRISTAL low-carbon construction-type steel. Samples of this steel were at first subjected to TAT, and subsequently analysed. The data acquired via the experimental analyses were further used to develop the Johnson-Cook constitutive equation, which was subsequently applied to numerically simulate the TAT experiment according to the real laboratory conditions. Finally, the experimental results were correlated to the simulated ones to validate the applicability and reliability of the developed material model.

## 2. Materials and Methods

The work is divided into three mutually connected stages. In the first one, TAT is performed experimentally to determine proper coefficients for the rheological law. The second part of the manuscript is then focused on verifying the correctness of these values using numerical simulations by a finite element code. Last but not least, the third part of the manuscript is devoted to practical verification of the validity of the designed Johnson–Cook (J–C) model. The verification of the J–C model is based on simulating a real manufacturing process with consideration of the designed J–C model.

### 2.1. Taylor Test, Theoretical Background

**Figure 1:**
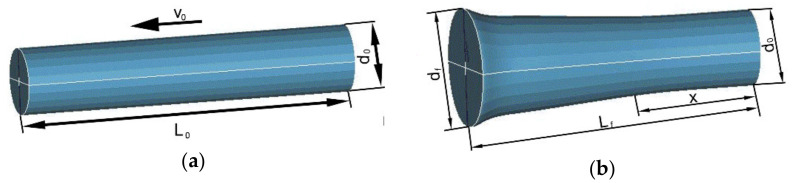
Depiction of TAT testing specimen: before TAT (**a**); after TAT (**b**).

The compressive Taylor anvil test (TAT), first presented in 1948 [27], is advantageously used to investigate behaviours of materials characterised with high imposed strains at high strain rates, i.e., strain rates about 10^3^ s^−1^ [28]. TAT is designed to simulate real industrial conditions of loading and can favourably be used to test bulk metallic specimens of simple geometries. During TAT, a cylindrical testing specimen of density *ρ*, initial length *L*_0_, and initial diameter *d_0_* is shot using expanding air with the initial speed *v*_0_, typically varying between 30 and 240 m·s^−1^, on a rigid impact plate (see Figure 1a for a schematic depiction of a TAT testing specimen). The impact plate can also be a high-strength rod or dynamometer, on which the elastic deformations and impact forces develop and the result of impact of the testing specimen with the rigid surface are detected. Immediately after the collision of the testing specimen with the rigid surface, the acting compressive force creates elastic and plastic waves, which begin to spread across the specimen. The elastic wave, spreading within the material at the speed of sound, bounces back from the free end of the specimen and returns as a tensile wave. The elastic wave can perform multiple bouncing from a surface, or interface. In a certain location within the specimen, both the waves interact. The typical shape of a TAT tested specimen is depicted in Figure 1b, where *L_f_* is the final length of the specimen, *d_f_* is the deformed diameter of the impacted forehead of the specimen, and *X* is the length of its undeformed cylindrical part.

### 2.2. Experimental

**Figure 2:**
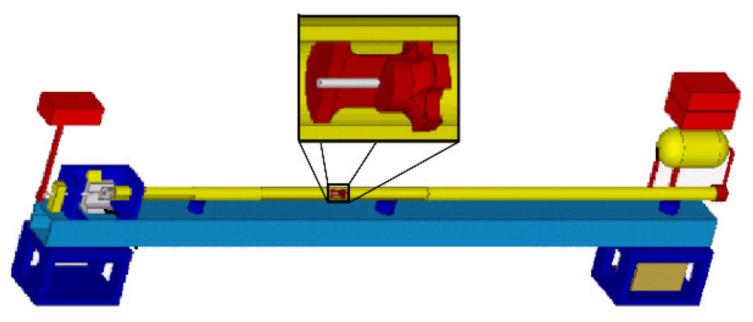
Schematic depiction of experimental apparatus for TAT.

For the presented study, TATs were performed using a pneumatic gun assembled and located at the Technical University of Brno (Brno, Czech Republic). A schematic depiction of the experimental TAT assembly is depicted in Figure 2. The impact rate for all the tested specimens was 235.8 m·s^−1^.

Cylindrical samples of TRISTAL low-carbon BCC-type steel fabricated by Moravia Steel a.s. (Třinec, Czech Republic) with the initial length, *L*_0_ = 25 mm and initial diameter *d*_0_ = 5 mm were used as testing specimens. The chemical composition of TRISTAL steel is summarised in Table 1. The specimens were fabricated from a hot-rolled product via slow machining with cooling, special care was given to parallelism of both the faces, which were finally polished mechanically on grinding machines (Struers GmbH, Willich, Germany). All the specimens were fabricated with the maximum deviation of ±0.05 mm (measured via the SOMET ČSN 251420 micrometre (SometCZ, Bílina, Czech Republic)). After testing, the dimensions of the tested specimens were carefully measured using the SOMET ČSN 251420 micrometre. The entire geometry of the specimen was scanned using a CCD camera (Celestron, Torrance, CA, USA) with the step of 0.1 mm and subsequently evaluated via image analysis available within the LUCIA Forensic software (Laboratory Imaging s.r.o., Prague, Czech Republic). Finally, the curve characterising the geometry of the tested specimen was transferred into a graph using Microsoft Excel software (Microsoft, Redmond, WA, USA).

To enable detailed characterisation of the effects of TAT on the structure and morphology of the TRISTAL testing specimens, initial spheroidising annealing at 700 °C for 24 h in an electric furnace was performed for all the samples. For structure characterisation via optical microscopy (OM), an Olympus optical microscope was used (Olympus, Tokyo, Japan). Further detailed structure observations were performed using scanning and transmission electron microscopies (SEM and TEM), for which Lyra 3 XMU scanning electron microscope (SEM, Tescan, Brno, Czech Republic) and JEM-2100 transmission electron microscope (TEM, JEOL, Tokyo, Japan) operating at 200 kV were used. The specimens were prepared for the microscopic analyses via combinations of manual grinding on SiC papers and polishing using diamond polishing pastes.

Mechanical testing of the samples was ensured via detailed hardness measurements. These were performed using the ZHV10 Vickers hardness testing machine (Zwick Roell CZ s.r.o., Brno, Czech Republic). Evaluation of the measured data and creation of 3D graphics was subsequently performed using the LUCIA Forensic software.

### 2.3. Numerical Simulation

The numerical simulation of TAT was performed using the ANSYS LS Dyna software (Ansys Inc., Canonsburg, Pennsylvania, USA). The deformable body of the testing specimen was defined by a hexagonal mesh with 474 747 elements and 496 679 nodes in total. No re-meshing was used. The initial temperature for TAT was set as 23 °C. The Johnson–Cook rheological law (Equation (1)) was used to define the TAT simulation. The parameters *σ*_0_, *B*, *n*, *C*, *m* of Equation (1) are determined experimentally in the presented work (as further described in the Section 3).

(1) $$\sigma =\left(\sigma_{0}+B\varphi^{n}\right)\left(1+C\ln\frac{\dot{\varphi }}{{\dot{\varphi }}_{0}}\right)\left(1-T^{*m}\right)$$

where *σ*_0_ is static flow stress, *B* is work hardening coefficient, *φ* is strain rate, *n* is work hardening exponent, *C* is strain rate sensitivity, *m* is the thermal softening coefficient, and T* is homological temperature defined in the range 0 < T* < 1 by Equation (2)

(2) $$T^{*}=\frac{T-T_{0}}{T_{m}-T_{0}}$$

where *T*_0_ is the reference temperature (at static flow stress *σ_0_ = σ*), and *T_m_* is the melting temperature.

## 3. Results

### 3.1. Structure Analyses

**Figure 3:**
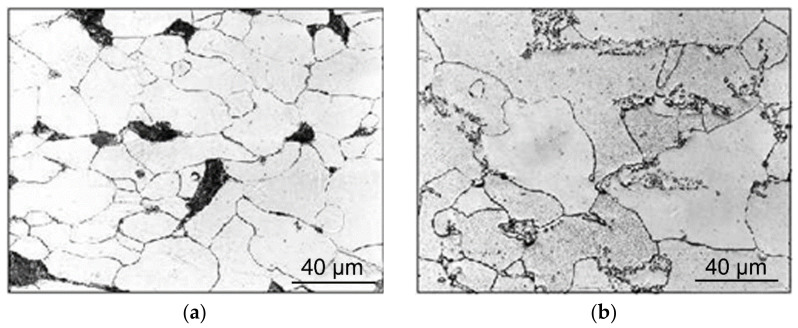
OM images of structure of TRISTAL steel: before spheroidising annealing (**a**); after spheroidising annealing (**b**).

The primary analyses of the TRISTAL steel specimens were focused on the observations of their structures. The structure of the steel was originally ferritic with the presence of perlitic particles, which primarily occurred at triple points (see Figure 3a for the original TRISTAL structure). The occurring perlitic colonies were subsequently transformed to globular cementite particles via the initial spheroidising annealing (see Figure 3b for the heat-treated TRISTAL structure).

**Figure 4:**
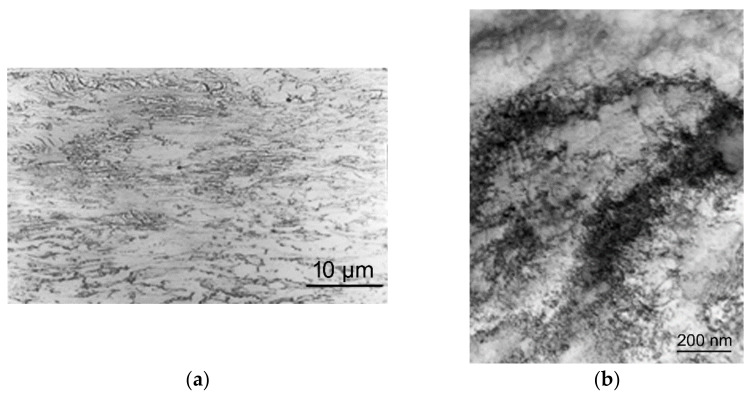
SEM image of the structure of impacted forehead of tested specimen, axial longitudinal section (**a**); TEM image of dislocations within deformed grains within structure of impacted forehead of tested specimen, axial longitudinal section (**b**).

Subsequent detailed structural analyses of the tested specimens performed via SEM and TEM confirmed that the structure in the impacted forehead of the tested specimen was heavily deformed. Figure 4a depicts an SEM image taken from the axial longitudinal section through the impacted specimen, heavily deformed grains can clearly be observed. Figure 4b then shows a detailed TEM image showing a high density of dislocations within the deformed grains.

### 3.2. Hardness Measurements

**Figure 5:**
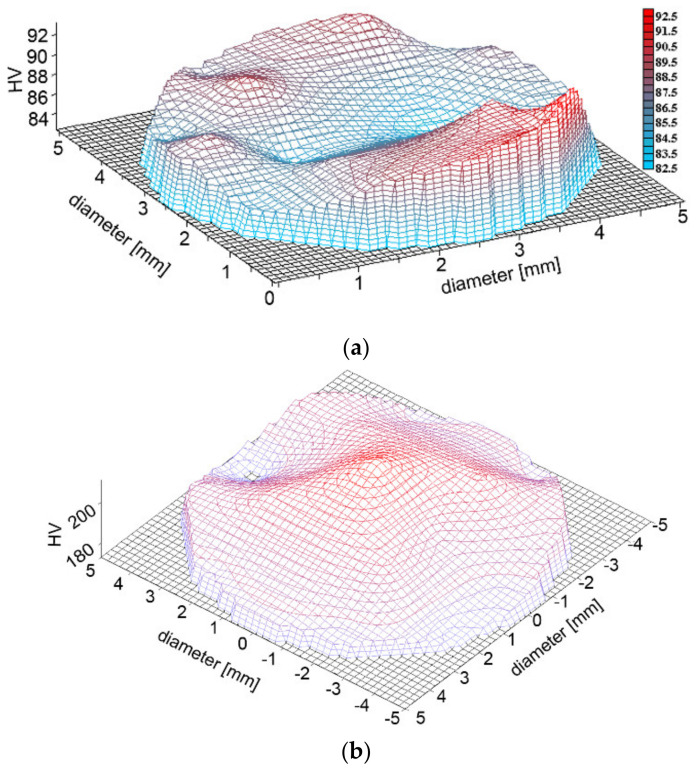

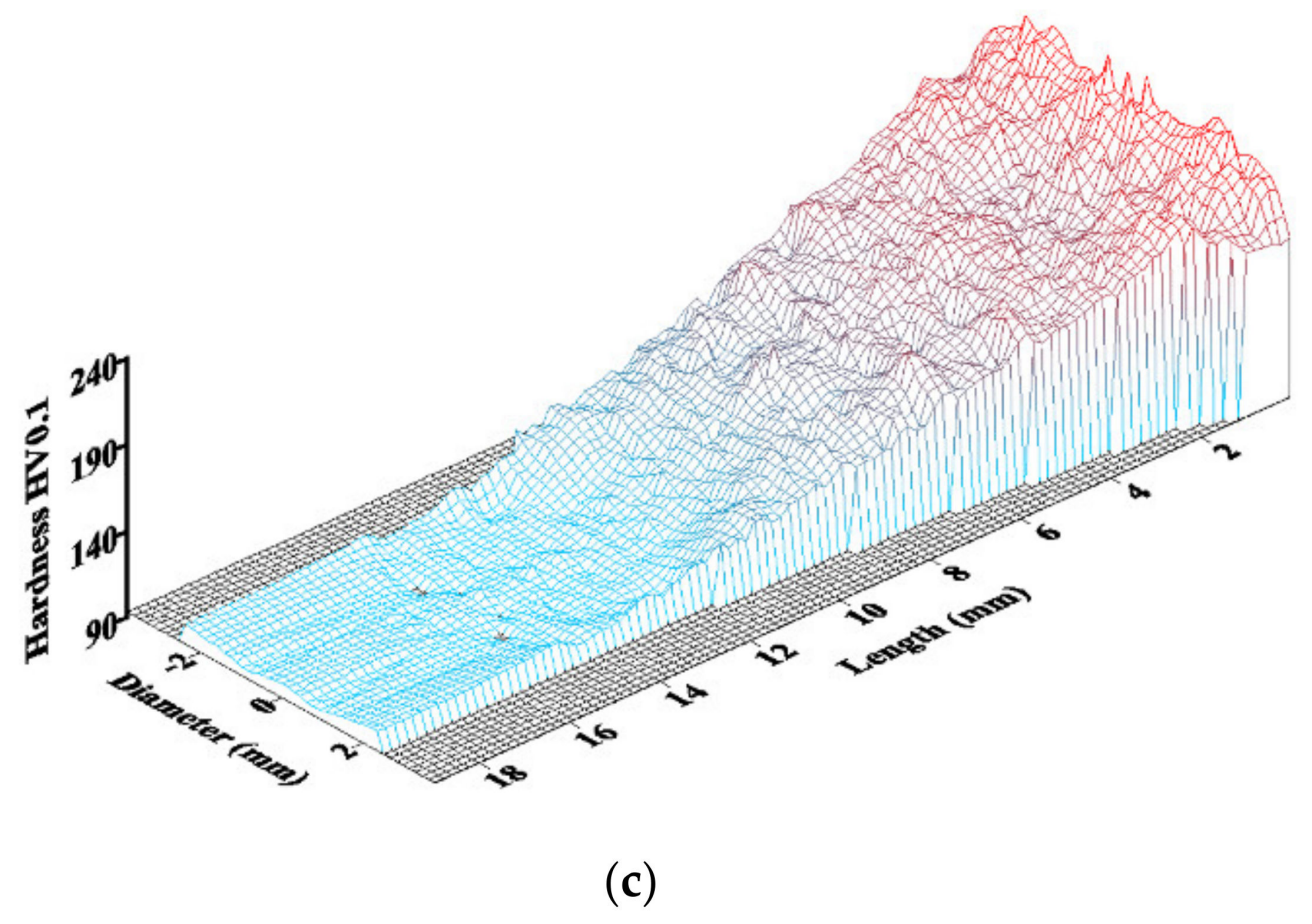
3D hardness maps: cross-section of the front surface of the original testing specimen (**a**); cross-section of impacted forehead of tested specimen after TAT (**b**); axial longitudinal section of tested specimen after TAT (**c**).

The original TRISTAL structure subjected to the initial spheroidising annealing (Figure 3b) exhibited an average hardness of 88 HV. After TAT, the maximum hardness values measured within the tested specimen increased to 205–235 HV. The cross-sectional 3D hardness map for the front surface of the original testing specimen is depicted in Figure 5a, whereas the cross-sectional 3D hardness map for the impacted forehead of the tested specimen is depicted in Figure 5b. Last but not least, the 3D map acquired along the axial longitudinal section of the tested specimen after TAT is depicted in Figure 5c.

### 3.3. TAT Specimen Analyses

The subsequent analyses focused on observing the geometry of the tested specimen were at first performed experimentally, then using image analysis methods, and finally using numerical simulations. Mutual correlation of all the results subsequently enabled verification of the computed values of the J–C constitutive equation parameters.

**Figure 6:**
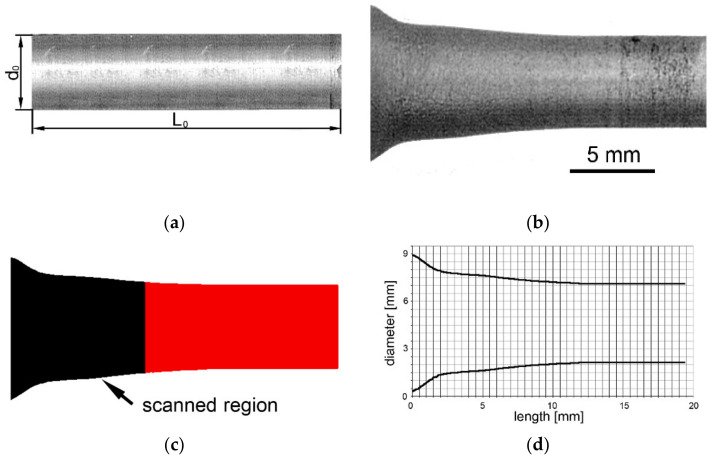
Testing specimen before TAT (**a**); tested specimen after TAT (**b**); acquired image of the surface of the tested specimen (**c**); final geometrical curve characterising tested specimen after TAT (**d**).

The geometry of the original specimen before TAT is depicted in Figure 6a (see Section 2.2 for the exact dimensions), whereas Figure 6b shows the deformed geometry of the tested specimen. Figure 6c depicts the filled contour of the tested specimen being acquired by the CCD camera. Finally, Figure 6d shows the final curve characterising the dimensions of the tested specimen after TAT.

**Figure 7:**
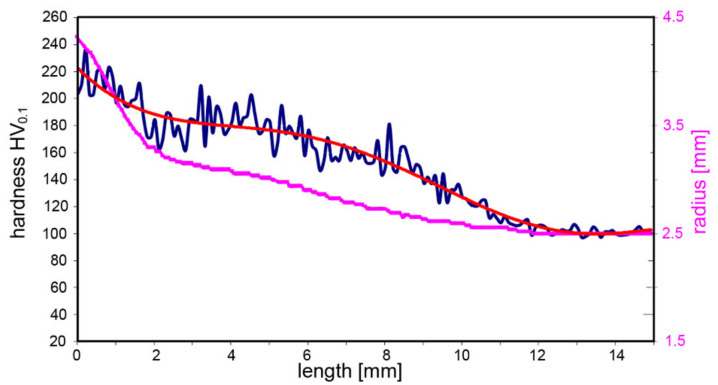
Geometry of tested specimen in correlation with HV0.1 hardness values.

Image analyses put in correlation the geometry of the tested specimen with the measured hardness values. A graphical depiction of the dependence of HV hardness values on the geometry of the specimen after TAT shown in Figure 7 documents that the hardness values correlated with the geometrical shape of the specimen.

### 3.4. Dynamic Flow Stress Analysis

Another important parameter necessary to enable reliable computation of the J–C constitutive equation for the tested specimens is the dynamic flow stress, the curves for which were computed using mathematical formulas after Meyers [29], Wilkinson–Guinan [29], Taylor [30], and Taylor for low impact velocities [31]. The dependence of dynamic flow stress on the impact velocity is approximated by multinomials.

**Figure 8:**
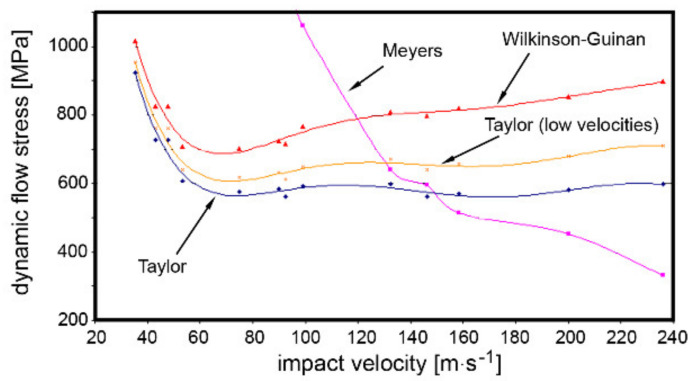
Dependences of dynamic flow stress on impact velocity for tested specimen calculated after selected equations.

Figure 8 depicts the curves calculated from the individual mathematical formulas. As can be seen, the dynamic flow stress increased rapidly with decreasing impact velocity under the value of ~50 m·s^−1^ for the Wilkinson–Guinan and both the Taylor curves. Above the impact velocity of ~50 m·s^−1^, both the dependencies calculated after Taylor exhibited character close to a linear dependency. Despite the fact that the Wilkinson–Guinan curve exhibited a character similar to the Taylor curves up to the impact velocity of 50 m·s^−1^, above this value, the Wilkinson–Guinan dynamic flow stress increased gradually with increasing impact velocity. Last but not least, the dynamic flow stress curve calculated after Meyers exhibited a steep increase with decreasing impact velocity below the value of ~120 m·s^−1^ and subsequent gradual decrease with increasing impact velocity above 120 m·s^−1^.

### 3.5. Finite Element Analyses

Having experimentally investigated the structure, geometry, and dynamic behaviour of the TRISTAL testing specimens, numerical simulations were performed and optimised to determine the ideal parameters of the J–C constitutive equation.

**Figure 9:**
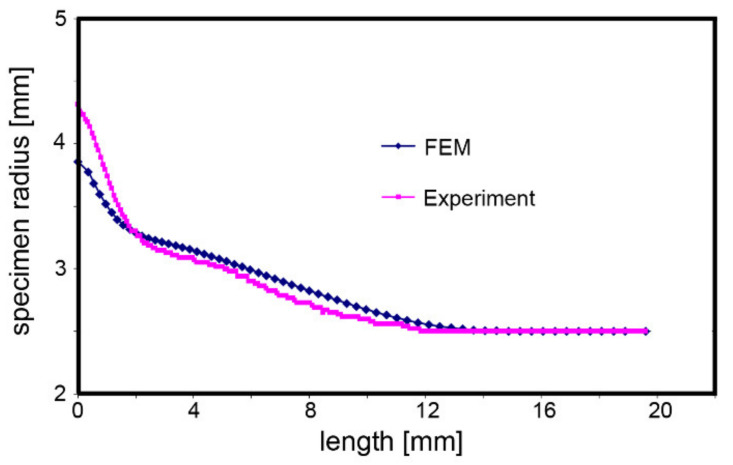
Comparison of experimentally acquired and numerically predicted results of tested specimen geometry (impact velocity 235.8 ms^−1^).

At first, the experimentally acquired geometry of the tested specimen was correlated with the geometry of the deformed specimen from the numerically simulated TAT. Both the specimen geometries are compared in Figure 9. The figure clearly shows a good correlation of both the sets of results as their maximum deviation was between 5% to 8%.

**Figure 10:**
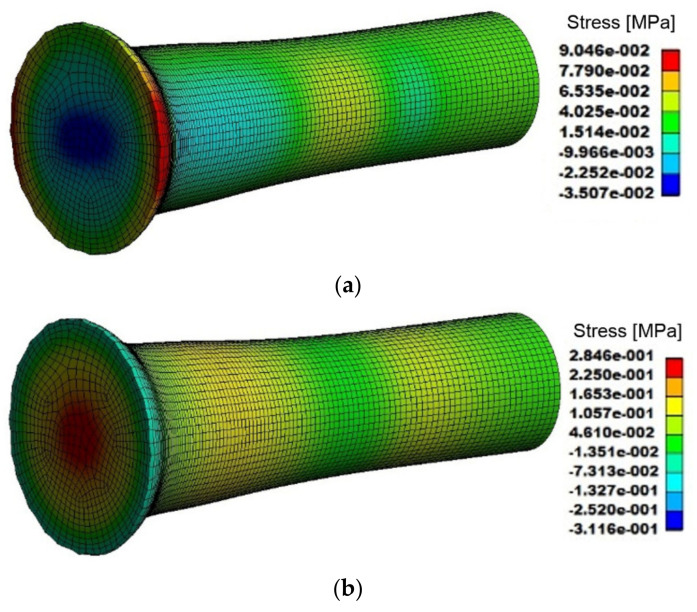

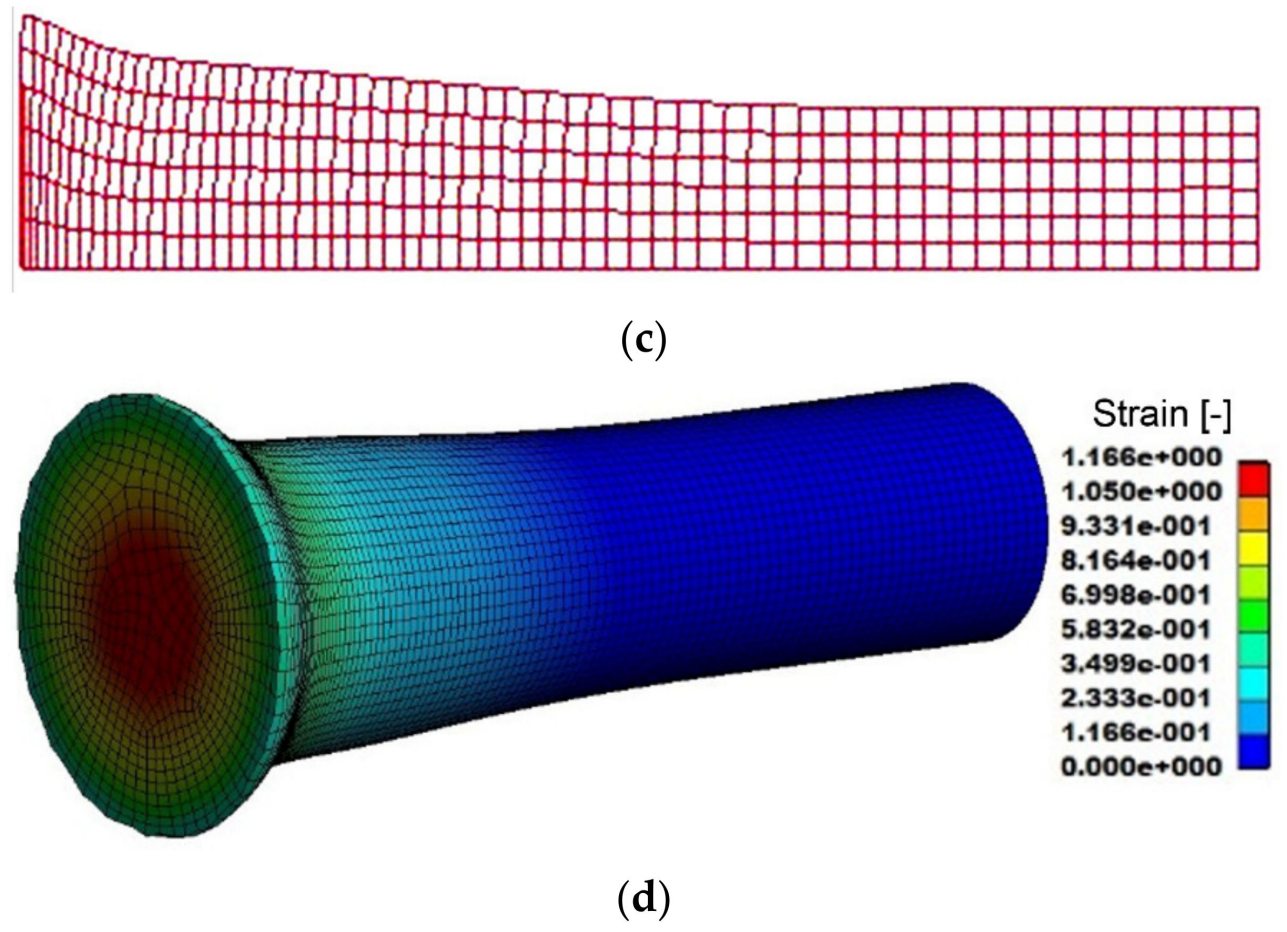
Numerically predicted distributions of: YZ stress component (**a**); compressive stress (**b**); 0.5 mm × 0.5 mm superimposed grid (axial longitudinal cut through tested specimen) (**c**); effective imposed strain (**d**).

Selected numerically predicted parameters and their distributions throughout the tested specimen after TAT are further depicted in Figure 10a–d. Figure 10a depicts the distribution of YZ stress components throughout the tested specimen after TAT, whereas Figure 10b depicts the distribution of compressive stress within the tested specimen. Figure 10c then shows half of the longitudinal cut through the tested specimen after TAT, a 0.5 mm × 0.5 mm grid through which was superimposed to demonstrate the deformation of the impacted forehead. Finally, Figure 10d depicts the values and distribution of effective imposed strain within the tested specimen.

**Figure 11:**
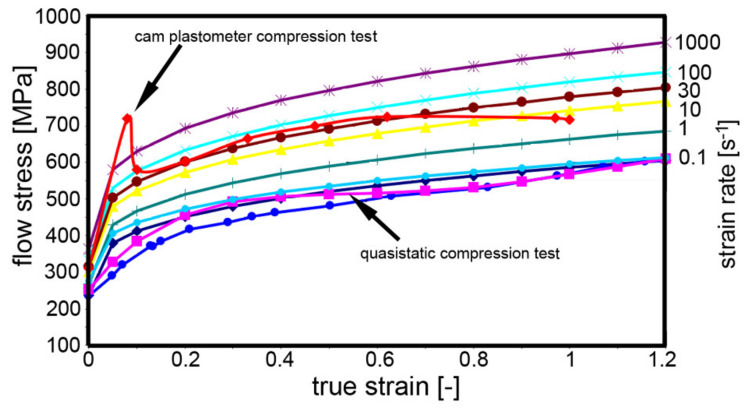
Flow stress curves for TRISTAL steel for temperature of 23 °C.

The final analysis performed to optimise the parameters of the J–C equation was the determination of the flow stress, i.e., stress-strain curves, in dependence on temperature and strain rate based on the comparison of the experimentally acquired and predicted data. The curves were determined for a room temperature of 23 °C, and the strain rates varied in the range from 0.1 to 1 000 s^−1^. The stress-strain curve derived based on the J–C equation for the strain rate of 0.1 s^−1^ was compared to the curve acquired via a standard quasi-static compression test (strain rates 10 s^−1^ and 30 s^−1^), and with the curve acquired via a compression test performed on a cam plastometer. The final comparison of the acquired stress-strain curves is depicted in Figure 11.

### 3.6. Determination of Parameters for Johnson–Cook Model

Having performed the optimisation of the material model via putting in correlation the experimentally acquired and numerically predicted data, the mathematical characterisation of the model was performed via determining the parameters of the Johnson–Cook constitutive equation. A summary of the material constants for the Johnson–Cook equation for TRISTAL low-carbon steel is depicted in Table 2.

### 3.7. Experimental Verification of Developed Johnson–Cook Model

**Figure 12:**
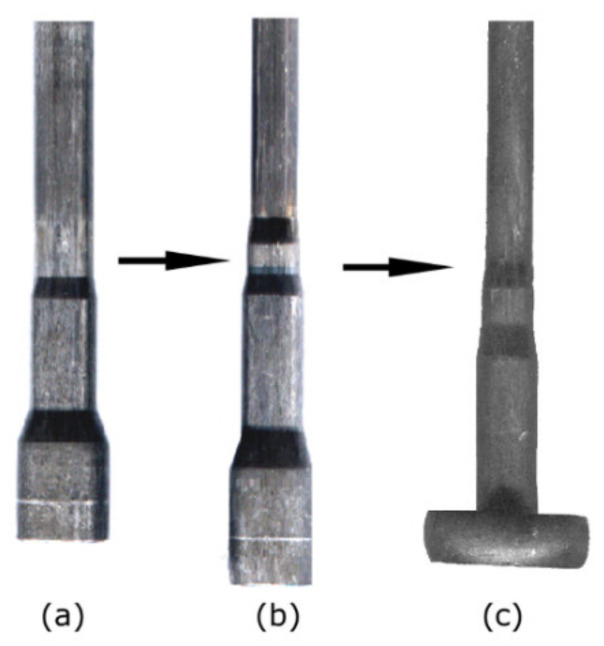
Three-step production process of fabrication of automobile component shaft via automatic sequential machine: pre-processing (**a**); direct extrusion (**b**); stamping (**c**).

TRISTAL steel is widely used in the automotive, typically to fabricate specific components, such as brake boosters, snub rolls for disk brakes, or members of transmissions and control units. During fabrication of such components via cold bulk forming using automatic sequential machines, the values of strain rates reach up to 1000 s^−1^ (see Figure 12 for an example of a component fabricated via sequential processing on automatic sequential machines).

Practical verification of the J–C material model developed for the TRISTAL steel based on the TAT experiment and subsequent numerical simulation was carried out via performing two consequent production steps of direct extrusion (step depicted in Figure 12b), and subsequent stamping (step depicted in Figure 12c). The used production machine was a TPZD 25 automatic sequential machine (Šmeral Brno a.s., Brno, Czech Republic) with rigid top and bottom anvils, an upstroke limit of 330 mm, and a maximum stroke rate of 60 min^−1^.

**Figure 13:**
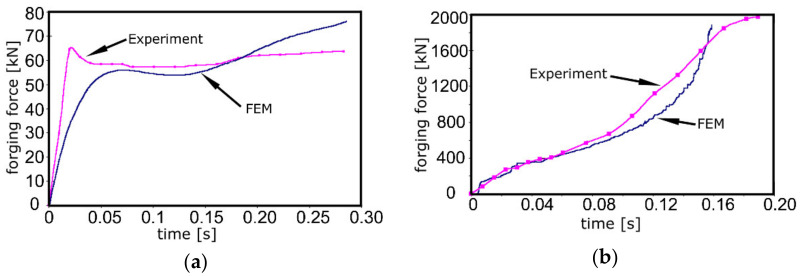
Development of forging forces in time during sequential processing: direct extrusion (**a**); stamping (**b**).

Figure 13a,b depict the dependences of forging forces on processing time for both the production steps, i.e., for direct extrusion and stamping, respectively. Figure 13a shows that the forging force exhibited a steep increase at the beginning of extrusion. This increase was followed by a mild drop and a subsequent gradual increase, which was eventually slightly higher for the simulated curve. The maximum experimental forging force for this production step was 64.5 kN, whereas the maximum simulated forging force was 77 kN. On the other hand, the development of the forging force during the processing step of stamping was significantly different (Figure 13b). Both the experimental and predicted forging force curves exhibited an increasing trend with increasing processing time. The maximum experimentally measured forging force for this processing step was 1976.7 kN, whereas the maximum numerically simulated forging force was 1890 kN.

## 4. Discussion

The first part of the study was focused on the detailed characterisation of the effects of the performed TAT on the structure and properties of the TRISTAL testing specimens. To be able to characterise the effects of TAT with the exclusion of the effects of previous deformation history, initial spheroidising annealing at 700 °C for 24 h in an electric furnace was performed for all the specimens. This type of annealing was selected as it is the most commonly used initial heat treatment for such types of steel intended for cold forming in the industry, applied to increase their formability [32]. The structure analyses showed that the perlitic colonies present in the original semi-product (Figure 3a) were successfully transformed to globular cementite particles in the heat-treated testing specimens (Figure 3b). This fact also positively affected the hardness of the specimens, as the average HV value of the heat-treated steel was 88 HV. The decreased hardness goes hand in hand with the increased ability of the steel specimens to plastically deform, which favourably increased the possibility to perform a detailed characterisation of the effects of TAT on their geometry, even for impact rates exceeding 200 m·s^−1^

The image analysis of a specimen after TAT revealed that significant changes in its geometry occurred to the depth of 12 mm from the impacted forehead. Additionally, the tested specimen evidently increased its diameter in the region in the vicinity of the impacted forehead at the expense of its total length (Figure 6d). The specimen’s region exhibiting changes in the geometry also featured a significant increase in hardness; besides the geometrical changes, one of the most notable effects of the significant plastic deformation occurring during TAT was the change in hardness. The HV values within the tested specimen were the highest at the impacted forehead, in its axial region in particular. The development of hardness further corresponded to the observed geometrical changes. In other words, the longer the distance from the impacted forehead along the specimen’s axis, the lower the hardness values. A similar trend was observed also across the cross-section of the tested specimen at the impacted forehead, the further the location from the axis of the tested specimen, the lower the hardness values. However, Figure 7 depicts that the overall increase in the hardness values was not only observed in the front bulge but within the entire volume of the tested specimen. The supplementary detailed structure analyses confirmed that the structure in the (vicinity of the) impacted forehead of the tested specimen was heavily deformed, which confirmed the occurrence of deformation hardening, which manifested as the increased hardness values.

Numerical predictions were further performed and optimised to determine the parameters of the J–C constitutive equation. For these purposes, we correlated the geometries of the experimentally tested and numerically simulated specimens, as well as investigated the predicted distributions of the most important stress-strain parameters and compared them to the experimentally acquired data on the behaviour and properties of the tested specimens. The experimental and predicted specimen geometries exhibited satisfactory correlation, which provided a solid basis for the subsequent comparison of the experimental and predicted mechanical behaviours.

The stress-strain data predicted via the simulation documented by Figure 10a–d showed that the highest values of all the parameters were detected in (the vicinity of) the impacted forehead of the tested specimen. The YZ stress component was the highest on the periphery of the impacted forehead, which corresponded to the experimentally observed geometrical changes of the specimen, as well as to the predicted material flow documented by the deformed superimposed grid shown in Figure 10c. The YZ stress component gradually decreased across the cross-section of the forehead towards the axis of the specimen, along which it was the lowest. The surface of the remaining cylindrical part of the tested specimen also exhibited slightly increased values of the YZ stress component, when compared to the axial region.

Contrary to the YZ stress component, compressive stress was the highest along the axis of the tested specimen and decreased towards its periphery. This also corresponds to the observed geometrical changes of the specimen and the results of material flow prediction. During the impact on the rigid plate, the specimen was compressed in the axial direction by the effect of the impact force. In the peripheral regions of the specimen, the material flow was not limited by any surrounding material volume and thus could compensate the effect of the impact force by changing its trajectory from the axial direction towards the surrounding free space (see the deformed grid in Figure 10c). However, towards the axis of the specimen, the material volume increased and for the axial region of the specimen, the surrounding material volume was the highest and thus also the most limiting regarding material flow. For this reason, the material flow in the axial region of the tested specimen was aggravated, which resulted in the high observed compressive stress values in this region.

The distribution of the effective imposed strain correlated to the distribution of compressive stress, as the highest values of effective strain were predicted in the axial region of the impacted forehead of the tested specimen. This finding corresponds to the significant presence of dislocations within the grains in this region, as documented by Figure 4b, as well as to the distribution of experimentally measured hardness, which also exhibited the highest values in the axial region of the impacted forehead (Figure 5b).

Optimisation of the parameters of the numerical simulation finally led to the determination of the parameters of the J–C equation, which were summarised in Table 2. The final equation was subsequently used to simulate a real sequential production process to validate the reliability of the developed model. The initial steep increase in the forging forces for direct extrusion (Figure 13a) was most probably connected to the high effective imposed strain in this processing step. Firstly, the steel was subjected to elastic loading and exhibited elastic deformation up to the point at which the forging force started to affect the grain boundaries. Releasing the accumulated energy by the development of plastic deformation correlated to the occurring mild drop in the forging force [33]. Continued loading of the steel component imparted gradual hardening of the material, which resulted in a gradual increase of the forging force. During the extrusion process, the experimentally measured forging force was generally slightly higher than the simulated one. However, in the final stage of extrusion, the simulated forging force increased and was finally higher than the experimentally measured one. The final difference can be explained by the fact that in the simulation, the anvil was defined to be absolutely rigid and thus, the final forging force was higher. The development of the forging force during stamping was different (Figure 13b). In other words, both the real and predicted forging force values were gradually increasing with increasing forging time. This phenomenon was partially caused by the filling of the anvil cavity with the stamped material, but also by the gradual deformation hardening of the steel [34,35].

## 5. Conclusions

The study was focused on the determination of the parameters of the Johnson–Cook constitutive equation for commercial TRISTAL low-carbon steel, widely used in the automotive industry, via experimental observations and subsequent numerical predictions. The results of optimised numerical simulations corresponded well with the experimentally acquired ones. The presented work led to the following conclusions:

- The predicted distribution of stress-strain parameters was in accordance with the deformed geometry and hardness of the experimental specimen;
- *n* hardening exponent and *m* strain rate sensitivity coefficient change at a value of effective strain of ~0.50;
- Optimised parameters of the Johnson–Cook equation for TRISTAL steel were *n* = 0.3 and *m* = 0.72;
- The results are not affected by friction-predicted geometry corresponded to the experiment when negligible friction was applied (due to ultra-high strain rate);
- The final verification of the material model performed via real production on TPZD25 automatic sequential machine, documented satisfactory correlation of predicted and experimental results.

## Figures and Tables

**Table 1:** Chemical composition of TRISTAL steel.

Element	C	Mn	Si	P	S	Cr	Ni	W	Cu
(wt.%)	0.1	0.38	0.07	0.012	0.008	0.05	0.04	0.01	0.1

**Table 2:** Parameters of developed Johnson-Cook constitutive equation.

Parameter	A [MPa]	B [MPa]	C [-]	*n* [-]	m [-]
value	273	391	0.051	0.3	0.72

## Data Availability

The original data supporting the research is not publicly available but the data that is not confidential is available on request from the corresponding author.
